# Clonidine stimulates force of contraction via histamine H_2_ receptors in the human atrium

**DOI:** 10.1007/s00210-023-02635-x

**Published:** 2023-07-25

**Authors:** Joachim Neumann, Steffen Pockes, Laura J. Humphrys, Denise Mönnich, Lisa Forster, Uwe Kirchhefer, Britt Hofmann, Ulrich Gergs

**Affiliations:** 1 Institute for Pharmacology and Toxicology, Medical Faculty, Martin-Luther-University Halle-Wittenberg, Magdeburger Straße 4, D-06097 Halle (Saale), Germany; 2 Institute for Pharmacology and Toxicology, Medical Faculty, Martin-Luther-University Halle-Wittenberg, Magdeburger Straße 4, D-06112 Halle (Saale), Germany; 3 Institute of Pharmacy, University of Regensburg, Universitätsstraße 31, D-93040 Regensburg, Germany; 4 Institute for Pharmacology and Toxicology, University Hospital Münster, Westfälische Wilhelms-University, Domagkstraße 12, D-48149 Münster, Germany; 5 Department of Cardiac Surgery, Mid-German Heart Center, University Hospital Halle, Ernst Grube Straße 40, D-06097 Halle (Saale), Germany

**Keywords:** Clonidine, Human atrium, Mouse atrium, Histamine H2 receptor

## Abstract

Clonidine has various clinical effects mediated by agonism of α_1_- or α_2_-adrenoceptors and the blocking of hyperpolarization-activated-nucleotide-gated pacemaker channels (HCN). It is unknown whether clonidine can also stimulate human cardiac histamine H_2_ receptors (hH_2_Rs). We used isolated electrically stimulated left and spontaneously beating right atrial preparations from mice overexpressing the hH_2_R specifically in the heart (H_2_-TG), and spontaneously beating right atrial preparations of guinea pigs for comparison. Moreover, we studied isolated electrically stimulated muscle strips from the human right atrium. Clonidine (1, 3, and 10 µM) increased force of contraction in isolated left atrial preparations from H_2_-TG mice. In contrast, clonidine reduced the spontaneous beating rate in right atrial preparations from H_2_-TG. Clonidine raised the beating rate in guinea pig right atrial preparations. Clonidine failed to increase the force of contraction but reduced beating rate in wild-type litter mate mice (WT). In WT, histamine failed to increase the force of contraction in left atrial preparations and beating rate in right atrial preparations. Clonidine (10 µM) increased the force of contraction in isolated human right atrial preparations. The positive inotropic effect in the human atrium was attenuated by cimetidine (10 µM). Clonidine increased the beating rate of the isolated spontaneously beating guinea pig right atrium and acted as a H_2_R partial agonist. Furthermore, clonidine showed binding to the guinea pig H_2_R (100 µM) using HEK cells in a recombinant expression system (p*K*_i_ < 4.5) but hardly to the human H_2_R. These data suggest that clonidine can functionally activate cardiac human H_2_R.

## Introduction

Clonidine, 2-[(2,6-dichlorphenyl)imino]imidazoline, was developed in the 1960s as a drug against swollen noses from the common cold (decongestant: Kobinger 1978, Kobinger 1984). Clonidine was effective in dog models of nose swelling and was planned to enter the clinic for this application (Kobinger 1978). At that time, clonidine’s decongestant action was thought to occur via stimulation of nasal vasoconstrictive α_2_-adrenoceptors (Kobinger 1978). By accident, it was found that clonidine was readily absorbed from nose drops, could pass the blood brain barrier, and was very potent and effective in reducing blood pressure and reducing heart rate in human volunteers (Kobinger 1978).

In canine studies, clonidine was shown to act on α_2_-adrenoceptors in the central nervous system and to inhibit the sympathetic outflow from the brain into the peripheral organs (Kobinger 1978). This reduced sympathetic outflow and noradrenaline levels in the peripheral vasculature and peripheral resistance fell, translating into a reduction in blood pressure (Kobinger 1978). Later, clonidine was shown to be an agonist at poorly defined central imidazoline-receptors, which might alternatively or additionally explain the anti-hypertensive effects of clonidine (Likungu et al. 1996).

**Fig. 1:**
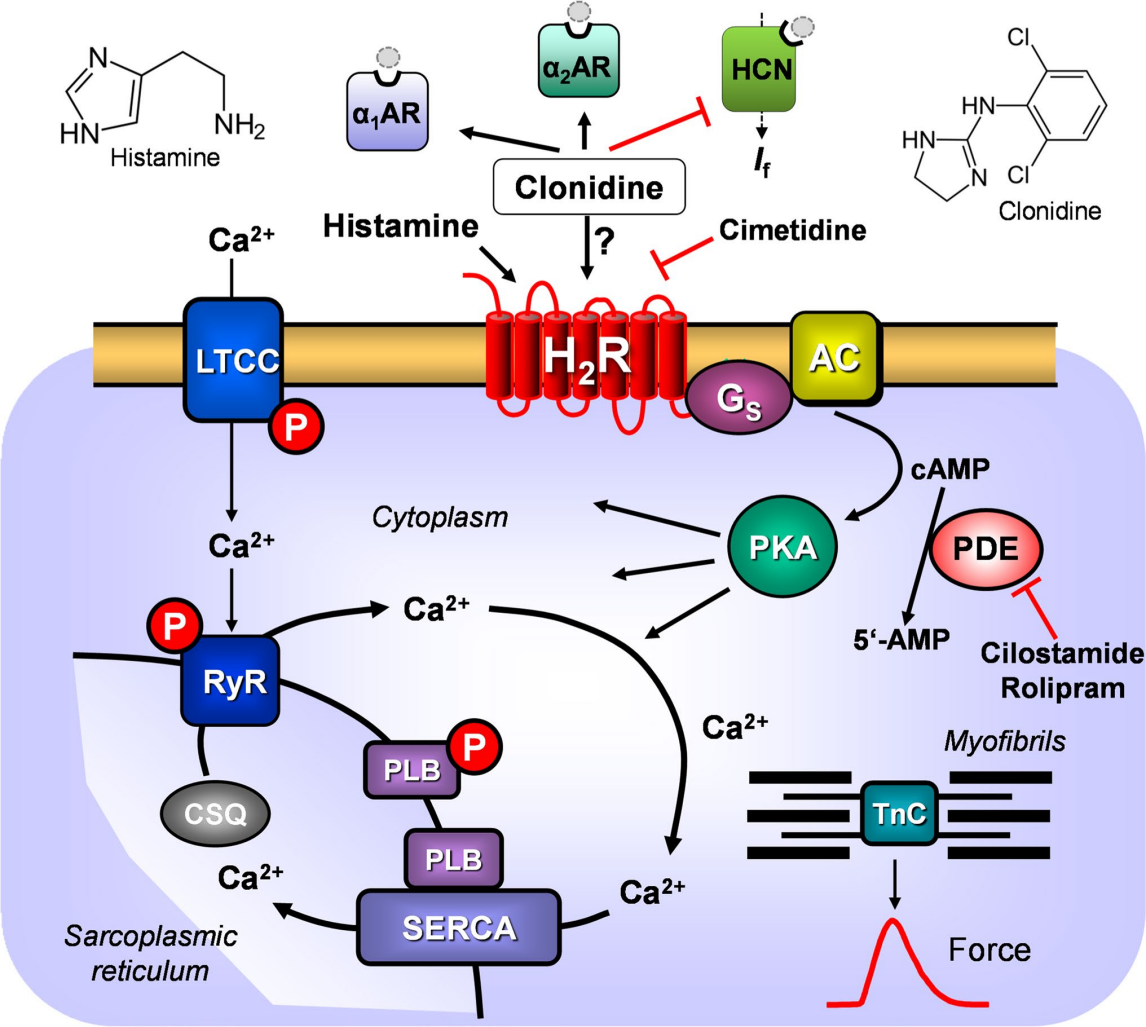
Scheme: Hypothetical human cardiac mechanism of action of clonidine. Stimulation of histamine H_2_ receptors (H_2_R) leads to enhanced levels of cAMP via stimulatory GTP-binding proteins (Gs) in cardiomyocytes because the activity of adenylyl cyclases (AC) is augmented. This cAMP could lead to increased activity of cardiac regulatory proteins via an elevation of the activity of cAMP-dependent protein kinases (PKA). PKA can phosphorylate the L-type Ca ion channel, increasing its open state and leading to more inflow of trigger Ca into the cell. This trigger Ca releases by action of the ryanodine receptor further Ca from the sarcoplamic reticulum (SR). This Ca binds an activates myofilaments to generate force (red curve). In relaxation, Ca is pumped again in the SR by means of SERCA. In the SR, Ca is bound to calsequestrin (CSQ). The relaxation is enhanced if PKA has phosphorylated phospholamban (PLB) or the inhibitory subunit of troponin (TnI). The cAMP is degraded and inactivated by phosphodiesterases (PDEs) within the cells. PDE-inhibitors like cilostamide or rolipram increase the levels of cAMP in the cell. Clonidine might stimulate α1- or α2-adrenoceptors (AR) or H_2_R or might inhibit hyperpolarization activated cyclic nucleotide channels (HCN). Cimetidene inhibits the H_2_R

Clonidine induced positive inotropic effects in isolated electrically driven rabbit papillary muscles (Schümann and Endoh 1976). These effects were blocked by prazosin and were therefore classified as α_1_-adrenoceptor mediated (Schümann and Endoh 1976) (Fig. 1).

Clonidine also directly inhibits cardiac hyperpolarization-activated-cyclic-nucleotide–gated pacemaker channels (HCN: Knaus et al. 2007a, b; Tanguay et al. 2019) (Fig. 1). When clonidine inhibited HCN-channels in the isolated beating animal atrium, a negative chronotropic effect ensued (wild-type mouse: Knaus et al. 2007a, b).

In addition, clonidine can also stimulate histamine H_2_ receptors (Fig. 1). In the heart, all four known histamine receptor subtypes (H_1-4_R) have been described at an RNA-level and/or protein-level (review: Neumann et al. 2021a, human heart: Matsuda et al. 2004). However, species, regional and cellular differences in histamine receptor function exist in the heart (review: Neumann et al. 2021a). In the mouse heart, a direct histamine receptor mediated inotropic or chronotropic effect is missing. Likewise in the rat, dog, and cat, inotropic effects of histamine were found to be indirect via release of endogenous catecholamines (Laher und McNeill 1980a, b). In the guinea pig heart, there are regional differences: H_2_Rs mediate positive chronotropic effects in both the perfused heart and isolated right atrium, but inotropic effects in the left atrium are solely H_1_R mediated (review: Neumann et al. 2021a, b, c, d, e). In the human atrium and ventricle, in contrast, H_2_Rs have been identified, and these can mediate the positive inotropic effects of histamine (review: Neumann et al. 2021a, b, c, d, e). In order to establish an animal model for the human (h)H_2_R in the heart, we have generated and characterized a transgenic mouse that overexpressed the hH_2_R only in the heart (H_2_-TG). In the atrium of H_2_-TG, histamine exerts positive inotropic and chronotropic effects but not in the wild-type litter mate mice (WT) atrium (Gergs et al. 2019).

In spontaneously beating atria, clonidine exerted a concentration-dependent positive chronotropic effect that was completely blocked by cimetidine (McCulloch et al. 1980; Verma and McNeill 1977a, b). Clonidine was less effective than histamine in these right atrial guinea pig preparations, and thus clonidine could be a partial agonist at H_2_Rs there (McCulloch et al. 1980; Verma and McNeill 1977a, b). In contrast to these publications, there is also conflicting evidence that clonidine fails to affect contractility (force or beating rate) in the guinea pig atrium (Rubio et al. 1982). However, in isolated perfused spontaneously beating guinea-pig hearts, clonidine also increased the cAMP-content (Verma and McNeill 1977a, b). This increase in cAMP was blocked by additionally applied burimamide, a H_2_R antagonist, arguing for a H_2_R-mediated effect of clonidine in the guinea pig heart (Verma and McNeill 1977a, b).

We tested here the main hypothesis that clonidine is an agonist at human cardiac H_2_Rs. To this end, we studied the effect of clonidine on atrial preparations from H_2_-TGs on the force of contraction and beating rate. Moreover, in a translational approach, we also studied the effect of clonidine in isolated human atrial preparations on the force of contraction and interrogated the receptor involved.

## Materials and methods

### Transgenic animals

The investigation conformed to the Guide for the Care and Use of Laboratory Animals published by the National Research Council (2011). Animals were maintained and handled according to approved protocols of the animal welfare committees of the University of Halle-Wittenberg, Germany, and the University of Regensburg, Regensburg, Germany. Here, transgenic mice with cardiac myocyte-specific overexpression the human H_2_R (H_2_-TG) were used. Cell-specific overexpression was achieved through the mouse cardiac α-myosin heavy chain promoter. The generation and characterization of this transgenic mouse model were described before (Gergs et al. 2019). All experiments were done with adult H_2_-TG mice and wild-type (WT) littermates of both sexes were used.

### Contractile studies on the isolated guinea pig right atrium (spontaneously beating)

Hearts were rapidly removed from guinea pigs and the right atrium was quickly dissected and set up isometrically in the Krebs–Henseleit solution under a diastolic resting force of approximately 5 mN in a jacketed 20 mL organ bath at 32.5 °C as previously described (Pockes et al. 2018). The bath fluid (composition [mM]: NaCl, 118.1; KCl, 4.7; CaCl_2_, 1.8; MgSO_4_, 1.64; KH_2_PO_4_, 1.2; NaHCO_3_, 25.0; glucose, 5.0; sodium pyruvate, 2.0), supplemented with (RS)-propranolol (0.3 μM) to block β-adrenergic receptors, was equilibrated with 95% O_2_ and 5% CO_2_. Experiments were started after 30 min of continuous washing and an additional equilibration period of 15 min. Two successive concentration response curves (CRCs) for histamine displayed a significant desensitization of 0.13 ± 0.02 (*N* = 16 control organs). This value was used to correct each individual experiment. For agonists, two successive concentration–response curves were generated: the first for histamine (0.1–30 μM) and the second for the agonist of interest. Additionally, the sensitivity to 316 μM cimetidine was checked at the end of each H_2_R agonist CRC, and a significant reduction of frequency was observed. The relative potency of the agonist under study was calculated from the corrected pEC_50_ difference (ΔpEC_50_). pEC_50_ values are given relative to the long-term mean value for histamine (pEC_50_ = 6.16) determined in our laboratory (pEC_50_ = 6.16 + ΔpEC_50_). Data were analyzed by nonlinear regression and were best-fitted to sigmoidal concentration–response curves using Prism 9.5.0 software (GraphPad, San Diego, CA).

### Contractile studies in mouse atrium

In brief, the right or left atrial preparations from the mice were isolated and mounted in organ baths as previously described (Gergs et al. 2019). The bathing solution of the organ baths contained 119.8 mM NaCI, 5.4 mM KCI, 1.8 mM CaCl_2_, 1.05 mM MgCl_2_, 0.42 mM NaH_2_PO_4_, 22.6 mM NaHCO_3_, 0.05 mM Na_2_EDTA, 0.28 mM ascorbic acid, and 5.05 mM glucose. The solution was continuously gassed with 95% O_2_ and 5% CO_2_ and maintained at 37 °C and pH 7.4. Spontaneously beating right atrial preparations from mice were used to study any chronotropic effects.

The drug application was as follows. After equilibration was reached, clonidine was cumulatively added to left atrial or right atrial preparations to establish concentration–response curves. Then, where indicated, carbachol was cumulatively applied to the preparations. Finally, after the response stabilized and without any washout, 1 µM isoprenaline was added to test whether the efficacy of clonidine was comparable to the maximum response to β-adrenergic stimulation.

### Contractile studies on human preparations

The contractile studies on human preparations were done using the same setup and buffer as used in the mouse studies (see the “Contractile studies in mouse atrium” section). The samples were obtained from 3 male and 2 female patients, aged 53–76 years (mean ± SD: 68.6 ± 9.6 years). The diagnoses of the patients were NYHA II–III and CCS II–III. Our methods used for atrial contraction studies in human samples have previously been published and were not altered in this study. This study in patients complies with the Declaration of Helsinki and has been approved by the local ethics committee (hm-bü 04.08.2005). Written informed consent was obtained for the use of right atrial tissues from patients undergoing cardiac surgery.

### Data analysis

Data shown are means ± standard error of the mean. Statistical significance was estimated using the analysis of variance followed by Bonferroni’s *t*-test or the chi^2^ test as appropriate. A *p*-value < 0.05 was considered to be significant.

### Radioligand competition binding

Radioligand competition binding experiments were performed as previously described by using the HEK293-SP-FLAG-hH_2_R cell line and [^3^H]UR-DE257 (*K*_d_ = 66.9 nM, *c* = 40 nM) or the HEK293T-CRE-Luc-gpH_2_R cell line and [^3^H]UR-KAT479 (*K*_d_ = 25 nM, *c* = 25 nM) (Pockes et al. 2018; Rosier et al. 2021; Baumeister et al. 2015; Tropmann et al. 2020). Ligand dilutions were prepared in L-15 with 1% BSA, and 10 μL/well was transferred to a flat-bottom polypropylene 96-well microtiter plate (Greiner Bio-One, Frickenhausen, Germany), as well as 10 μL/well of the respective radioligand. The cells were adjusted to a density of 1.25 × 10^6^ cells/mL, and 80 μL of the cell suspension was added to each well (total volume of 100 μL). All data were analyzed using GraphPad Prism 9 software (San Diego, CA, USA). The normalized competition binding curves were then fitted with a four-parameter logistic fit yielding pIC_50_ values. These were transformed into p*K*_i_ values using the Cheng–Prusoff equation (Cheng and Prusoff 1973).

### Drugs and materials

The drugs isoprenaline-hydrochloride, clonidine, prazosin, and cimetidine were purchased from Sigma-Aldrich (Germany). All other chemicals were of the highest purity grade commercially available. Deionized water was used throughout the experiments. Stock solutions were prepared fresh daily.

## Results

**Fig. 2:**
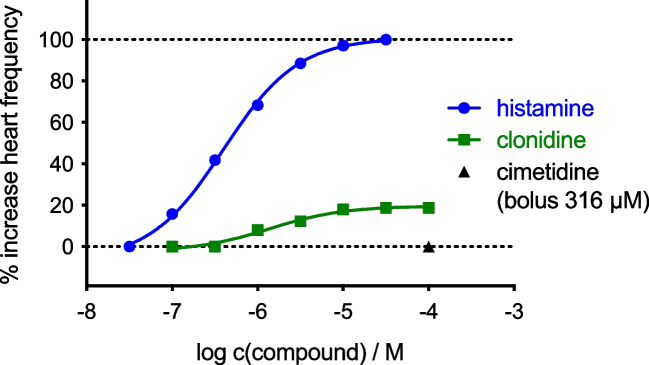
Representative concentration–response curves of histamine (reference) and clonidine including 316 μM bolus of cimetidine on the isolated spontaneously beating guinea pig right atrium (gpH_2_R). Displayed curves were calculated by endpoint determination. Number of individual experiments was four (*n* = 4)

Because cardiac effects of clonidine in guinea pigs are controversial in the literature, we first studied the effect of clonidine on the spontaneously beating rate in guinea pig right atria: clonidine exerted positive chronotropic effects in guinea pig right atrial preparations as a partial agonist (pEC_50_ = 5.45 ± 0.21; *E*_max_ = 0.19 ± 0.05; *n* = 4, mean ± SEM, Fig. 2) and these effects were antagonized by cimetidine (316 µM, Fig. 2). Hence, these data indicate that clonidine acts in the guinea pig heart as a partial agonist via the gpH_2_R.

**Fig. 3:**
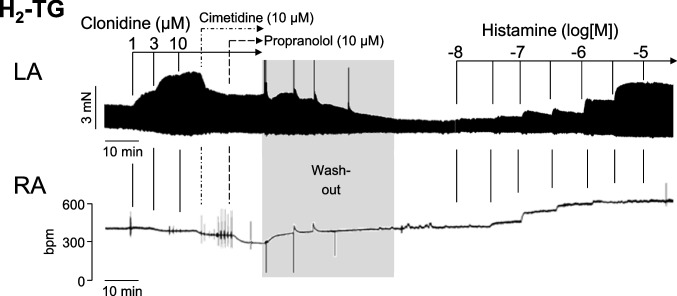
Original recording in mouse left atrial (top) and right atrial preparations from H_2_-TG. It becomes apparent that clonidine (left hand side) and histamine (right hand side) induced a time- and concentration-dependent positive inotropic effect in H_2_-TG. However, while histamine increased beating rate, clonidine reduced beating rate. Where indicated with an arrow first clonidine and then propranolol were added. Ordinates give force of contraction in milli Newton (mN, top) or beating rate in beats per minute (bpm). Horizontal bars give time in minutes (min)

**Fig. 4:**
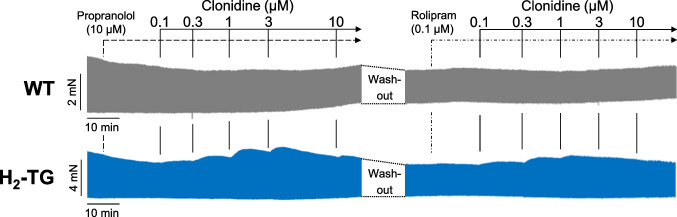
Original recording in mouse left atrial preparations from H_2_-TG (bottom) or WT (top). Clonidine induced a time- and concentration-dependent positive inotropic effect in H_2_-TG but not WT. On the right hand side, the experiment was repeated in the same muscle strips in the presence of rolipram. Ordinates: force of contraction in milli Newton (mN). Horizontal bars indicate time in minutes (min). Several changes of buffer in the organ bath are meant by “washout.” Cumulatively applied concentrations of clonidine are indicated by vertical lines in µM. Initially 10 µM propranolol was added to which clonidine was added. On the right hand side, first rolipram was given followed by clonidine

**Fig. 5:**
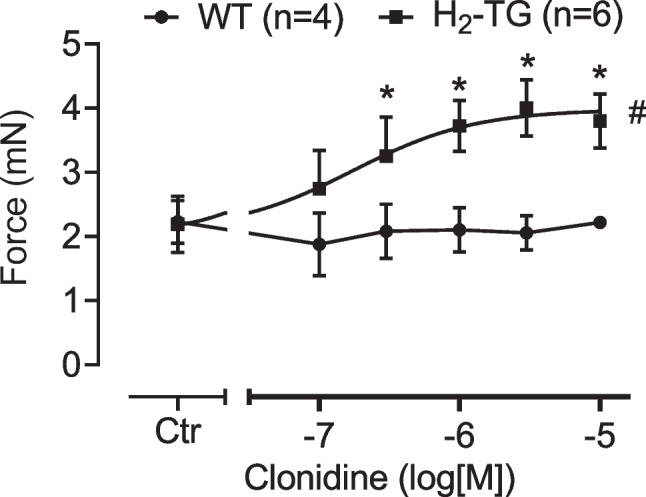
Summarized concentration–response curve for the effect of clonidine on force of contraction left atrial preparations from H_2_-TG and WT. Number in brackets gives number of experiments. Ordinate indicates force of contraction in milli Newton. Abscissa: Concentration of clonidine in logarithmic units in mole/liter (M). Ctr is pre-drug value. *p < 0.05 vs. Ctr; ^#^*p* < 0.05 vs. WT

Next, we studied cardiac effects of clonidine in mice. Clonidine concentration- and time-dependently increased force of contraction in left atrial preparations from H_2_-TG. This is depicted in an original recording from left atrial preparations from H_2_-TG in Fig. 3 (upper lane, left hand side). This effect of clonidine is lacking in WT (Figs. 4 and 5). In contrast, clonidine concentration-dependently reduced beating rate in right atrial preparations from H_2_-TG (Fig. 3, bottom). Subsequently applied cimetidine reduced force of contraction that had been increased by clonidine (Fig. 3, upper lane). To assess whether the effect of clonidine might be mediated via the release of noradrenaline, we also added 10 µM propranolol. However, although propranolol was able to reduce beating rate (lower lane in Fig. 3), propranolol failed to reduce the force of contraction any further (Fig. 3, upper lane). Thereafter, washout was performed (Fig. 3, middle) and histamine was cumulatively applied. This enabled us to see, in direct comparison, that histamine exerted a positive inotropic effect (Fig. 3, right hand side). Histamine was more potent but as efficacious as clonidine, suggesting that clonidine acts as full and not as a partial agonist. To test this, in separate experiments, we applied 300 nM histamine and thereafter increasing concentrations of clonidine (1, 3, and 10 µM). Here, clonidine did not decrease but instead increased histamine-stimulated force in the left atrium from H_2_-TG, consistent with the hypothesis that clonidine acted as a full agonist at H_2_R. Moreover, the time course of force generation in the left atrium was faster after histamine application than after clonidine application, where a slow increase in force was noted at each drug addition to the organ bath (Fig. 3, left versus right hand side).

Effects of clonidine on isolated right atrial preparations from H_2_-TG are depicted in Fig. 3 (lower lane). In contrast to the results in the guinea pig right atrial preparations (Fig. 2), clonidine failed to increase the beating rate and, in fact, reduced the beating rate in right atrial preparations from H_2_-TG (Fig. 3, upper lane). This phenomenon also occurred in WT, suggesting a common mechanism in both genotypes, potentially via the reported inhibitory action of clonidine on HCN-channels to decrease mouse heart beating rate in vivo (Knaus et al. 2007a, b).

Next, we reversed the mode of application. First, 10 µM propranolol was added, and then clonidine was cumulatively applied. Under these conditions (Fig. 4), a similar picture emerged as in Fig. 3: clonidine concentration- and time-dependently increased force of contraction in the left atrial preparations of H_2_-TG (Fig. 4 top, left hand side) but not in WT (Fig. 4 bottom, left hand side).

Furthermore, we studied the role of phosphodiesterase (PDE IV), the main phosphodiesterase in the mouse heart (Neumann et al. 2021b). To that end, atrial strips from WT and H_2_-TG were stimulated by 0.1 µM rolipram (Fig. 4, right hand side). Thereafter, clonidine was cumulatively applied. The potency of clonidine to raise force of contraction in H_2_-TG was not altered but clonidine was still ineffective in WT (Fig. 4 top, right hand side).

Data for the positive inotropic effect of clonidine are summarized in Fig. 5: we noted a concentration-dependent increase by clonidine in the force of contraction of H_2_-TG but no increase in the force of contraction of WT (Fig. 5).

**Fig. 6:**
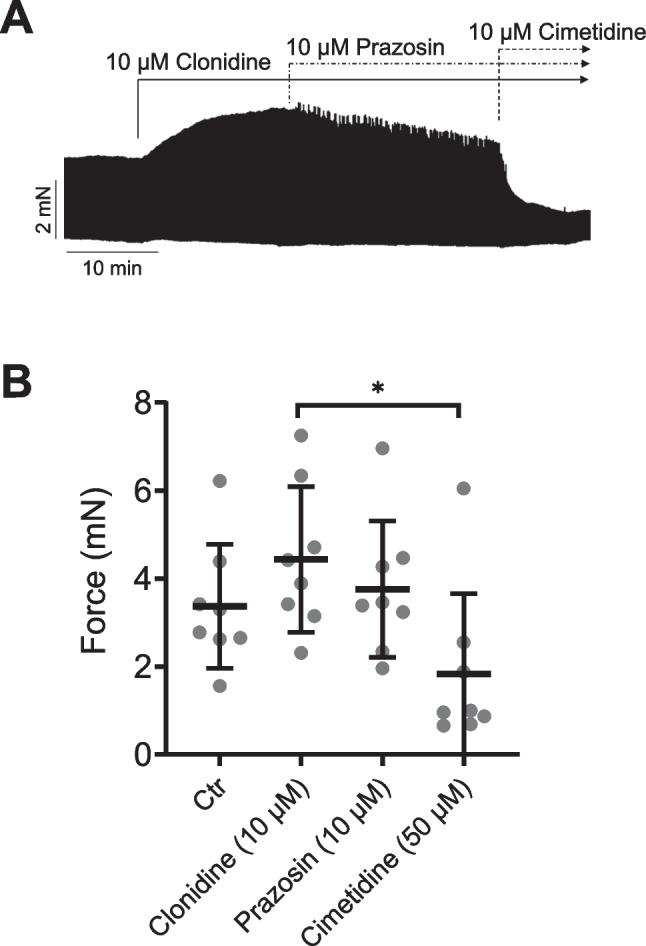
**A** Original recording: effect of clonidine alone or additional prazosin or additional cimetidine on force of contraction in isolated electrically stimulated right atrial preparations of human atrium. Ordinate force of contraction in mN (ordinate) in electrically stimulated human right atrial preparations. Abscissa gives time in minutes (min). **B** Summarized response for the effect of clonidine alone or in the additional presence of prazosine or Cimetidine. Ordinate: Force of contraction in milli Newton (mN). *p < 0.05 clonidine versus cimetidine. Eight preparations from five patients were analyzed

For translational knowledge, we wanted to understand how clonidine acted in the human heart. We used the same protocol in the human atrial tissue as with the mouse atrium. As seen in the original recording in Fig. 6A, clonidine at 10 µM increased force of contraction in a time-dependent manner. Additionally applied prazosin partially reduced the force of contraction. Force was decreased completely by additionally applied cimetidine, suggesting a H_2_R mediated effect (Fig. 6B). A small negative inotropic effect of prazosin is apparent, consistent with the ability of clonidine to stimulate α_1_-adrenoceptors. The main effect, however, was the reduction by cimetidine in the force of contraction, consistent with a stimulation of H_2_R by clonidine in human heart muscle strips (Fig. 6B).

**Fig. 7:**
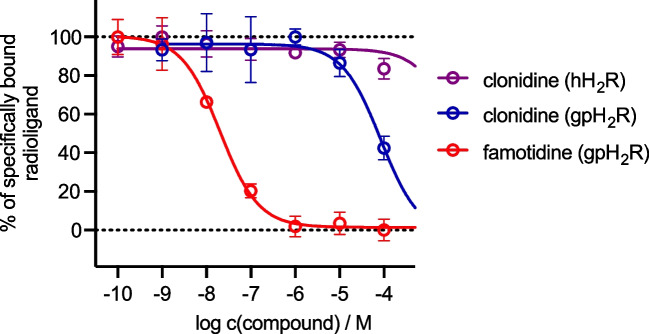
Displacement curves from representative radioligand competition binding experiments performed with clonidine, famotidine (reference), and [^3^H]UR-DE257 (*K*_d_ = 66.9 nM, *c* = 40 nM) using HEK293-SP-FLAG-hH2R cells or [.^3^H]UR-KAT479 (*K*_d_ = 25 nM, *c* = 25 nM) using HEK293T-CRE-Luc-gpH_2_R cells. Number of individual experiments was five at the hH_2_R (*n* = 5) and three at the gpH_2_R (*n* = 3)

We finally tested the binding behavior of clonidine at H_2_R. To that end, we studied various concentrations of clonidine in radioligand competition binding experiments at the human and guinea pig H_2_R. Here, clonidine bound to the guinea pig H_2_R (100 µM) using HEK cells in a recombinant expression system (p*K*_i_ < 4.5, *n* = 3). In contrast, clonidine did hardly bind to the human H_2_R (*n* = 5) (Fig. 7). In comparison, famotidine, a H_2_R antagonist, bound more potently than clonidine to guinea pig H_2_R.

## Discussion

### Main new findings

The main new findings in this report observe that clonidine acts as an agonist at human H_2_Rs. Species differences in the effects of clonidine on histamine receptors in the heart were reported between rabbits and guinea pigs when studying the force of heart contraction (Kenakin and Angus 1981). In a separate study, clonidine could stimulate adenylate cyclase activity in membranes from guinea pig hearts (Kanof and Greengard 1979) Clonidine was a partial agonist at the adenylate cyclase (Trist and Leff 1985). In our contraction experiments with H_2_-TG atria, clonidine was functionally a full agonist.

### Effects of clonidine on force of contraction in mouse left atrial preparations

In isolated perfused guinea pig hearts, clonidine increased left ventricular force of contraction via H_2_R (Csongrady and Kobinger 1974). The H_2_R antagonist burimamide antagonized the positive inotropic effect of clonidine in the isolated spontaneously beating guinea pig heart (Csongrady and Kobinger 1974). The response of clonidine was as efficacious as histamine but had a 25-fold decrease in potency (Csongrady and Kobinger 1974). This supports our findings in left atrial preparations from H_2_-TG where histamine was also more potent than clonidine. Again, clonidine exerted a concentration-dependent positive inotropic effect starting at 1 µM clonidine in isolated guinea pig papillary muscles, which was antagonized by cimetidine and thus was determined to be H_2_R mediated (Sanchez-Chapula 1981). This was accompanied by a shortening of the time of tension relaxation (Sanchez-Chapula 1981). The positive inotropic effects of clonidine were not affected by propranolol, prazosin, or an H_1_R antagonist in the guinea pig papillary muscle (Sanchez-Chapula 1981). Clonidine increased “slow potentials” in guinea pig papillary muscles at 500 nM and higher concentrations, indicative of an increase in the current through L-type Ca^2+^ channels (Sanchez-Chapula 1981, Fig. 1).

### Effects of clonidine in right atrial preparations

Clonidine did not increase the beating rate in the spontaneously beating guinea pig heart in a previous study (Csongrady and Kobinger 1974). Addition of the H_2_R antagonist burimamide revealed that clonidine exerted a negative chronotropic effect (Csongrady and Kobinger 1974). Interestingly, in our isolated beating guinea pig right atrial preparations, clonidine only exerted a positive chronotropic effect that is blocked by cimetidine (Fig. 2). One might question that clonidine might alternatively have increased the beating rate via alpha adrenergic receptors (Fig. 1) in the guinea pig right atrial preparations. We think this is unlikely, because by others reported that phenylephrine, an agonist at alpha and beta adrenoceptors, failed to increase the beating rate in isolated guinea pig atrial preparations via alpha-receptors but phenylephrine sole acted via ß-adrenoceptors (e.g., Nigro and Scivoletto 1983). Moreover, the positive inotropic effect of clonidine in guinea pig papillary muscles was blocked by cimetidine but not by 1 µM prazosin or 10 µM yohimbine (Sanchez-Chapula 1981).

In further studies in which clonidine was examined in radioligand competition binding experiments in a recombinant expression system at the human and guinea pig H_2_R, binding to the gpH_2_R (100 µM) but not to the hH_2_R was detected (Fig. 7). Of note, clonidine is an inverse agonist and burimamide is a neutral antagonist at the H_2_R (review Neumann et al. 2021a, b, c, d, e). Perhaps clonidine stimulated H_2_Rs but also blocked HCN-channels in the sinus node of the guinea pig heart, thereby stopping the action of H_2_Rs activation on the heart rate.

We would postulate that in the right atrial preparations, the stimulatory effect of clonidine on H_2_Rs is completely overwhelmed by a high sensitivity (higher than in the guinea pig right atrium) of HCN-channels for clonidine in the mouse right atrium. In our guinea pig right atrial preparations, the beating rate is stimulated by clonidine via H_2_Rs. In right atrial preparations from WT mice, clonidine reduced the beating rate. This finding is in agreement with work from others that clonidine reduces the rate of beating in isolated right atrial preparations from non-transgenic mice via inhibition of the current through HCN-channels (Knaus et al 2007a, b). Thereafter, in the same right atrium, histamine increased the beating rate in right atrial preparations from H_2_-TG via hH_2_Rs. In WT, clonidine likewise reduced the beating rate via HCN-channels, but histamine did not increase the beating rate because functional H_2_Rs receptors are lacking in WT preparations (Fig. 3, right hand side).

### Effects in human right atrial preparations

Our data show that clonidine acts via H_2_Rs in the human heart. It is already known that histamine acts in human and guinea pig hearts via H_2_Rs as the effects of histamine are blocked by cimetidine, a H_2_R antagonist. This holds true for the positive inotropic effects of histamine in the ventricle of guinea pig and man, and in the human right atrium (review: Neumann et al. 2021a, b, c, d, e). One other study failed to detect a positive inotropic effect of clonidine in isolated electrically driven human right atrial preparations (Ask et al. 1987). However, Ask et al. (1987) only studied 1 µM clonidine, while we used 10 µM clonidine. Based on our data of the action of clonidine on human H_2_Rs in left atrium of H_2_-TG, the lack of inotropic effect of clonidine in left atrium of WT and the fact that the positive inotropic effect of clonidine in the isolated human right atrium was attenuated by cimetidine, we would conclude that there is sound evidence for a direct stimulatory effect of clonidine on H_2_Rs in human cardiomyocytes.

### Clinical relevance

Peak plasma concentrations of clonidine under therapeutic dosage are low: 0.5 ng/mL (2.17 nM) (Buchanan et al. 2009). Moreover, clonidine is degraded by CYP2D6 (Claessens et al. 2010). Poisoning with clonidine leading to direct stimulation of cardiac H_2_Rs might occur if patients were given drugs that are inhibitors of CYP2D6 or if they express an inactive polymorphism of CYP2D6. In patients suffering on human heart failure, a decrease in heart rate and negative inotropic and lusitropic effects of clonidine have been reported (Azevedo et al. 1999). The negative inotropic effects were explained by a stimulation of α_2_-adrenoceptors by clonidine, which led to a reduced spillover of noradrenaline and translated into a reduction in the stimulation of cardio stimulatory β-adrenoceptors (Azevedo et al. 1999). The negative chronotropic effect of clonidine in the patients is probably mediated by central mechanisms and not by inhibition of the cardiac HCN-channels as the dosage was low.

### Limitations of the study

We did not have the opportunity to study contractility in the human ventricle or beating rate in human sinus node cells due to lack of access to that tissue. One can argue that clonidine might release histamine from the heart by stimulating, e.g., alpha_2_-adrenoceptors on cardiac mast cells. This released histamine itself would increase the force of contraction in H_2_-TG. One way to prove or refute this alterative mechanism would require to crossbreed mice with histidine decarboxylase knock out mice with our H_2_-TG to get double transgenic mice that do no produce endogenous histamine. This however is beyond the scope of the present study. Thus, at present, we cannot rule out that clonidine in the heart of mouse and man is, at least, in part also an indirect histaminergic agent. We did not perform binding studies in native cardiac tissue. Histamine receptors are present in probably all cells types found in the heart (e.g., Neumann et al. 2021a). One would expect that the binding of clonidine to H_2_Rs on cardiomyocytes explains its positive inotropic effect. We used the alpha-myosin heavy chain promoter to drive the overexpression of the human H_2_R. This promoter only drives overexpression in cardiomyocytes. Thus, we are convinced that we are measuring a functional effect of clonidine on H_2_R in mouse cardiomyocytes and not an effect on say H_2_R on endothelial cells that release a mediator to increase indirectly force of contraction. Therefore, on purpose we performed the binding to recombinant receptors in a cell line in order to get a homogenous cellular background.

## Conclusions

The present work indicated that in guinea pig right atrial preparations clonidine used H_2_R to mediate a positive chronotropic effect (Fig. 1). In transgenic mice that overexpress the H_2_R, clonidine used H_2_R to induce a positive inotropic effect (in left atrial preparations). In contrast, in mouse right atrial preparations, clonidine probably via HCN decreased the beating rate. In human right atrial muscle strips, clonidine increased force of contraction to a minor part via α_1_-adrenoceptors. The major part of the positive inotropic effect of clonidine on human right atrial muscle preparations, stimulate in vitro, was mediated via stimulation of human H_2_R.

## Data Availability

The data of this study are available from the corresponding author upon reasonable request.
